# Plasma-Like Culture Medium for the Study of Viruses

**DOI:** 10.1128/mbio.02035-22

**Published:** 2022-12-14

**Authors:** Mikhail V. Golikov, Birke Bartosch, Olga A. Smirnova, Olga N. Ivanova, Alexander V. Ivanov

**Affiliations:** a Engelhardt Institute of Molecular Biology, Russian Academy of Sciences, Moscow, Russia; b Université Claude Bernard Lyon 1, INSERM 1052, CNRS 5286, Centre Léon Bérard, Centre de recherche en cancérologie de Lyon, Lyon, France; Albert Einstein College of Medicine

**Keywords:** culture medium, metabolism, redox biology, virus

## Abstract

Viral infections attract more and more attention, especially after the emergence of novel zoonotic coronaviruses and the monkeypox virus over the last 2 decades. Research on viruses is based to a great extent on mammalian cell lines that are permissive to the respective viruses. These cell lines are usually cultivated according to the protocols established in the 1950s to 1970s, although it is clear that classical media have a significant imprint on cell growth, phenotype, and especially metabolism. So, recently in the field of biochemistry and metabolomics novel culture media have been developed that resemble human blood plasma. As perturbations in metabolic and redox pathways during infection are considered significant factors of viral pathogenesis, these novel medium formulations should be adapted by the virology field. So far, there are only scarce data available on viral propagation efficiencies in cells cultivated in plasma-like media. But several groups have presented convincing data on the use of such media for cultivation of uninfected cells. The aim of the present review is to summarize the current state of research in the field of plasma-resembling culture media and to point out the influence of media on various cellular processes in uninfected cells that may play important roles in viral replication and pathogenesis in order to sensitize virology research to the use of such media.

## INTRODUCTION

Viral infections have attracted increasing attention during the last decades due to many factors including the emergence in human populations of several zoonotic viruses such as severe acute respiratory syndrome-associated coronaviruses (SARS-CoV) 1 and 2 (1, 2), Middle East respiratory syndrome-associated coronavirus (MERS-CoV) (3), and monkeypox virus variants (4) as well as some highly pathogenic strains of influenza virus (5). The response to these events includes establishment of laboratory cell culture and animal models that will allow subsequent investigation of the virus life cycle and development of low-molecular-weight inhibitors and/or prophylactic vaccines (for examples, see references 6 to 8). It is not surprising that the major systems are based on *in vitro* cultivation of infected cells. Of course, such systems should reproduce various steps of viral life cycles and processes that are involved in the context of *in vivo* infections. Nevertheless, for decades virologists have been using not the cell lines corresponding to the target tissue the viruses infect but rather cell lines that ensure high-level replication. This is exemplified by numerous studies on SARS-CoV-2 and an array of other viruses that are being propagated in Vero or Vero E6 cells, cell lines that were established from a kidney of an African green monkey (7, 9), or on influenza virus that is passaged in Madin-Darby canine kidney (MDCK) cells obtained from a kidney of a cocker spaniel (10, 11). In recent years many laboratories have also been working with cancer or immortalized cells derived from the appropriate tissue/organ (i.e., A549 or Calu3 in the case of respiratory infections). However, immortalization or transformation is also known to affect the metabolic landscape of a cell and the activity of many signaling pathways (9). This drawback has been partially overcome by use of targeted differentiation of induced pluripotent stem (IPS) or progenitor cells, as well as by usage of primary cells derived from a patient’s organs (12, 13). One of the most notable examples is the use of hepatocyte-like cells obtained by differentiation of the liver progenitor HepaRG cell line; such cells can be infected with hepatitis B and delta viruses (HBV and HDV, respectively), although with moderate efficiency (8, 14). Use of primary cells obtained from a patient’s organs after transplantation or tumor resection for infection has many obstacles. Obviously, primary cells are not readily available for scientists, especially since noninvasive tests and laparoscopic surgeries are now generally applied, thus reducing the clinical need for transplantations or resections. In addition, primary cells have a high heterogeneity and do not always support replication of viruses. Moreover, primary cells in two-dimensional (2D) culture often undergo dedifferentiation leading to a loss of specific characteristics of the respective tissue (for example, see reference 15). The latter is frequently neglected by scientists who do not control the differentiation status throughout an experiment.

Another drawback of most *in vitro* infection systems is the usage of classical media such as minimal essential medium (MEM), Dulbecco’s modified Eagle’s medium (DMEM), DMEM–F-12 medium, and RPMI medium (16–19) that have profound impact on cell metabolism, redox status, and some signaling pathways and, as a result, on cell morphology and differentiation. When they were developed in the 1950s to the 1960s, the main goal of these media was to ensure a high rate of cell proliferation and biomass growth at low cost as well as avoidance of frequent replenishment of nutrients. The history of their development is described in an excellent review (20). Therefore, their formulations lack many physiological metabolites, whereas other key metabolites are present at nonphysiological levels (Table 1). These media support replication of a wide range of viruses at high levels and allow evaluation of compounds as directly acting antivirals. However, in the metabolomics/biochemistry field a growing number of reports show that classical media affect many metabolic pathways of a cell and modulate sensitivity to drugs that target host cell proteins. Therefore, other medium formulations were needed to avoid this pitfall.

**TABLE 1:** Comparison of nutrient concentrations in classical and novel media with their levels in human blood

Metabolite	Concn (μM) in:								
	DMEM	MEM	DMEM–F-12	RPMI 1640	Plasmax	HPLM	Neurobasal-A	SMEM	Human plasma^*a*^
Proteinogenic amino acids									
l-Alanine			50		510	430	22	510	427 ± 84
l-Arginine	398	265	699	1,148	64	110	398	64	114 ± 15
l-Asparagine			50	379	41	50	6	41	82 ± 7
l-Aspartic acid			50	150	6	20		6	21 ± 6
l-Cysteine			100		33	40	260		34 ± 10
l-Cystine	201	101	100	208	65	100		65	63 ± 28
l-Glutamate			50	136	98	80		98	97 ± 13
l-Glutamine	4,000	2,000	2,500	2,055	650	550		650	510 ± 118
Glycine	400		250	133	330	300	400	330	325 ± 127
l-Histidine	200	200	150	97	120	110	200	120	131 ± 37
l-Isoleucine	802	397	415	381	140	70	802	140	61 ± 19
l-Leucine	802	397	450	381	170	160	802	170	99 ± 12
l-Lysine	798	396	499	219	220	200	798	220	179 ± 58
l-Methionine	201	101	116	101	30	30	201	30	30 ± 6
l-Phenylalanine	400	194	215	91	68	80	400	68	78 ± 21
l-Proline			150	174	360	200	67	360	198 ± 65
l-Serine	400		250	286	140	150	400	140	160 ± 27
l-Threonine	798	403	449	168	240	140	798	240	128 ± 41
l-Tryptophan	78	49	44	24	78	60	78	78	55 ± 10
l-Tyrosine	398	199	214	111	74	80	398	74	55 ± 10
l-Valine	803	393	451	171	230	220	803	230	212 ± 61
Nonproteinogenic amino acids									
α-Aminobutyrate					41	20			
l-Citrulline					55	40		55	
l-Homocysteine					9				
4-Hydroxy-l-proline				153	13	20			
l-Ornithine					80	70		80	67 ± 15
l-Pyroglutamate					20				
Amino acid derivatives									
*N*-Acetylglycine					70	90			
l-Carnosine					6				
GSH				3	37	25			
Taurine					130	90		130	
Betaine					72	70			72 ± 22
Other components									
Acetate					42	40			42 ± 15
Acetone					55	60			54 ± 30
Acetyl carnitine					5				
Citrate					114	130			114 ± 27
Carnitine					46	40			46 ± 12
Creatine					37	40			37 ± 28
Creatinine					74	75			87 ± 19
Formate					33	50			33 ± 13
Fructose						40			
Galactose						60			
d-Glucose	25,000	5,556	17,490	11,101	5,560	5,000		5,560	4,971 ± 373
Glycerol					82	120			432 ± 100
2-Hydroxybutyrate					31	50			31 ± 8
3-Hydroxybutyrate					77	50			77 ± 66
3-Hydroxyisobutyrate					20				
Hypoxanthine			15		5	10			34 ± 10
Lactate					500	1,600			1,489 ± 371
Malonate						10			14 ± 1
Methyl acetoacetate					41				41 ± 36
Phenol red	40	29	23	14	25	14	22	25	
Pyruvate			500		100	50	227	100	35 ± 25
Succinate					23	20			
Uracil					2				
Urate					270	350			
Urea					3,000	5,000			6,075 ± 2,154
Uridine					3.00				
Linoleic acid			0.15						
Lipoic acid			0.51						
Putrescine			0.50						
Thymidine			1.51						
HEPES							10,924.37		
Inorganic salts									
Ammonium chloride					50	40			
Calcium chloride	1,802	1,802	1,051	21	1,800	2,350	1,802	1,800	
Calcium nitrate				424		40			
Magnesium chloride			301			480	814		
Magnesium sulfate	814	811	407	407	813	350		813	
Potassium chloride	5,333	5,333	4,157	5,333	5,330	4,100	5,333	5,330	
Sodium bicarbonate	44,048	26,188	29,024	23,810	26,191	24,000	26,190	44,050	
Sodium chloride	110,345	117,263	120,612	103,448	118,706	105,000	51,724	118,706	
Sodium phosphate	906	1,017	453	5,634	1,010	870	906	1,010	
Trace elements									
Ammonium metavanadate					0.0026				
Cupric sulfate			0.0052		0.0052				
Ferric nitrate	0.2475		0.12		0.12		0.25		
Ferric sulfate			1.50		1.04				
Manganous chloride					0.0002				
Sodium selenite					0.0289				
Zinc sulfate			1.50		1.50		0.67		
Vitamins									
*p*-Aminobenzoate				7.3		7.3			
Ascorbate					62.0				
d-Biotin			0.01	0.82	4.10	0.80			
Choline	28.6	7.1	64.1	21.4	7.1	21.5	28.6	7.1	15 ± 5
Folate	9.1	2.3	6.0	2.3	2.3	2.3	9.1	2.3	
*myo*-Inositol	40.0	11.1	70.0	194.4	11.1	194.3	40.0	11.1	
Niacinamide	32.8	8.2	16.6	8.2	8.2	8.2	32.8	8.2	
d-Pantothenic acid hemicalcium	8.4	2.1	4.7	0.5	4.2	1.1	8.4	2.1	
Pyridoxine	19.4	4.9	9.8	4.9	4.9	4.9	19.6	4.9	
Riboflavin	1.1	0.3	0.6	0.5	0.3	0.5	1.1	0.3	
Thiamine	11.9	3.0	6.4	3.0	3.0	3.0	11.9	3.0	
Vitamin B_12_			0.5019	0.0037	0.0050	0.0037	0.0050		

a Values taken from reference 57.

In the last decade several attempts to revise the composition of culture media were made, although none of them aimed to study viral infections. As such, the BrainPhys medium that was developed for cultivation of neuronal cells contains optimized levels of several amino acids and neuroactive ions (21). Another example is Spinner minimum essential medium (SMEM), which harbors amino acids, pyruvate, and vitamins at physiological levels and was used by the Tardito group for cultivation of breast cancer and glioblastoma multiforme cells (22). Note that these media were still lacking many important metabolites. The most advanced approach was presented by the Tardito and Sabatini laboratories that introduced two similar medium formulations, namely, human plasma-like medium (HPLM) (23) and Plasmax (24), which closely resemble human blood plasma. The rationale involved analysis of plasma composition from published databases and reconstitution of media with the metabolites that were present in plasma at levels above the selected threshold of 2 μM (24). As a result, the two media have almost the same composition. Although not much time has passed since their development, a series of papers reported that substitution of Plasmax or HPLM for classical media does change the status of redox metabolic pathways (data described below). Moreover, several successful examples demonstrate usage of these physiological media for molecular biology and, what is important for us, virology studies.

The first aim of this review is to summarize current knowledge on how classical media and their components affect the metabolism of uninfected cells and to show the pitfalls of their usage for cultivation of viruses. The second goal is to discuss how medium composition affects already-published data on cellular redox status and to speculate on how the use of physiological media may impact the interplay between pathogens, metabolism, and redox status. The third rationale is to summarize the scarce data from the use of these media for replication of viruses and to discuss why usage of Plasmax/HPLM is critical in virology. Although experimental data in this field of research are still scarce, and most findings come from the metabolomics/biochemistry field, we hope to draw attention to the importance of adapted cell culture media for obtaining physiologically relevant results.

## CHANGES IN CELL METABOLISM DURING VIRAL INFECTIONS: BRIEF DATA FROM CLASSICAL MEDIA

**FIG 1:**
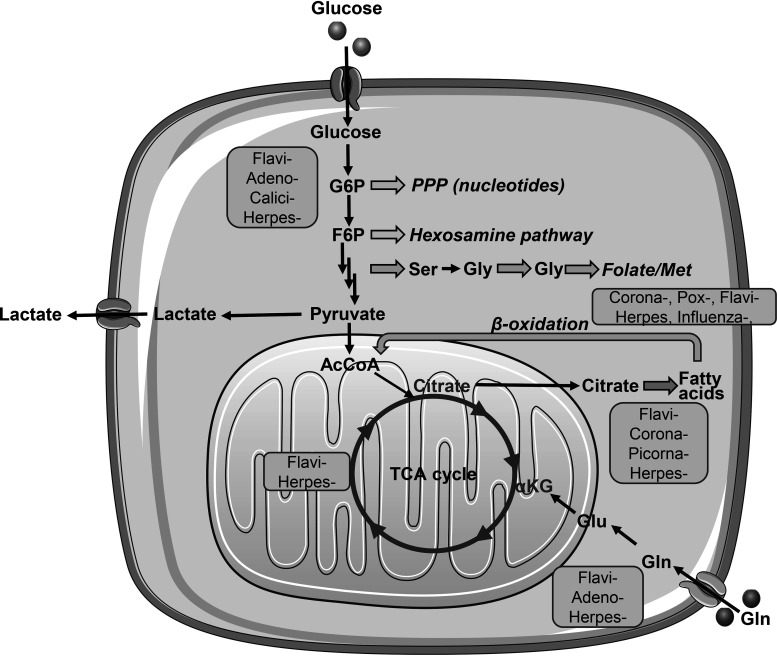
Scheme of central carbon metabolism. Glucose is converted into pyruvate via the glycolysis pathway, and the pyruvate either is converted into acetyl-CoA (AcCoA), a carbon donor of the tricarbonic acid (TCA) cycle, or is converted to lactate, and lactate is secreted from a cell. Glycolysis intermediates glucose-6-phosphate (G6P), fructose-6-phosphate (F6P), and fructose-1,6-bisphosphate serve as precursors for the pentose phosphate pathway that gives rise to nucleotides, the hexosamine pathway, and the *de novo* serine biosynthesis pathway. The latter is converted into glycine which feeds folate and later methionine cycles. Alternatively, the TCA cycle depends on glutamine that is converted into glutamate and then into α-ketoglutarate (αKG) and on β-oxidation of fatty acids. Fatty acid biosynthesis is initiated from citrate in the cytoplasm. Various viruses belonging to the *Flaviviridae*, *Herpesviridae*, *Coronaviridae*, *Adenoviridae*, *Caliciviridae*, *Poxviridae*, *Picornaviridae*, and other families interfere with these pathways.

As cell metabolism is tightly linked with cell growth and differentiation, and changes in its pathways are associated with development of an array of diseases, it is not surprising that the impact of viral infections on metabolic pathways have been studied by dozens of groups worldwide. There are several excellent reviews on the subject (25–29), so here we will present just a brief overview. It was shown that both acute and chronic viral infections interfere with central carbon metabolism by enhancing glycolysis and modulating mitochondrial respiration (Fig. 1). Upregulated anaerobic glycolysis, also referred to as the Warburg effect, supplies various anabolic pathways by which amino acids, sugars, and nucleotides are synthesized (25). These include the pentose phosphate pathway (PPP), the glucosamine pathway, and the *de novo* serine biosynthesis pathway that also gives rise to glycine and folate metabolism. The latter is tightly linked to synthesis of *S*-adenosylmethionine, which is widely used by various cellular methylases (30). The exact mechanisms by which viral pathogens activate glycolysis are species dependent. Zika virus of the *Flaviviridae* family upregulates expression of glycolytic enzymes and transporters via the AMP-activated protein kinase (AMPK) pathway (31), while noroviruses achieve this by activation of the phosphatidylinositol 3-kinase (PI3K)/AKT pathway (32), and adenoviruses and hepatitis C virus do this via the cMyc transcription factor (33–35). In most of these cases the resulting pyruvate does not feed the tricarbonic acid (TCA) cycle but is converted into lactate that is later exported from the infected cell.

To support mitochondrial respiration that is dependent on the Krebs cycle, infected cells often exhibit activated glutaminolysis, i.e., conversion of glutamine into α-ketoglutarate (αKG), a metabolite of the TCA cycle (34, 36, 37). The viruses achieve this by cMyc-dependent upregulation of glutamine transporters (SLC7A5 and SLC1A5) and glutaminase (GLS). Notably, enhanced anaerobic glycolysis and glutaminolysis could be considered a tumor-like phenotype (25).

Enhanced biosynthesis of fatty acids and lipids is another feature of viral infections. Fatty acids are synthesized from acetyl coenzyme A (AcCoA) that is produced in the cytoplasm by ATP-citrate lyase (ACLY) (38). AcCoA is converted into malonyl-CoA and then into various fatty acids by two-carbon elongation steps. These stages are catalyzed by the acetyl-CoA carboxylase 1 (ACC1) and fatty acid synthase (FASN), respectively. Their expression as well as expression of other proteins involved in lipid biosynthesis is regulated by the common lipogenic transcription factor sterol regulatory element-binding protein (SREBP) (38) that is activated by flaviviruses, herpesviruses, coronaviruses, and other pathogens (39, 40). Moreover, viruses promote transcription of fatty acid-biosynthetic enzymes via various signaling pathways such as AMPK or mTOR/SREBP, which controls expression of ACLY and ACC1 (41, 42) (in the case of human cytomegalovirus [HCMV]), or p38 mitogen-activated protein kinase (MAPK), which upregulates ACC1 (in the case of coxsackie virus B3) (43). Another strategy of modulation of FASN activity is scavenging this enzyme to the sites of virus replication, as shown in case of dengue virus (44). HCMV also promoted lipid biosynthesis by upregulating the expression of acetyl-CoA synthetase short-chain family member 2 (ACSS2), which synthesized AcCoA directly from acetate (45). These events lead to enhanced biogenesis of lipid droplets (46, 47), the endoplasmic reticulum (ER)-derived organelles that store neutral lipids (48) and often serve as sites for replication/virion assembly and budding of certain viruses (49–51).

Viruses also interfere with β-oxidation of fatty acids that is mainly localized in mitochondria. This process is initiated by import of fatty acid-CoA via carnitine palmitoyltransferase (CPT) and subsequent shortening of the carbon chain in multiple enzymes including mitochondrial trifunctional protein (MTP) (52). Respiratory viruses (influenza virus, SARS-CoV-2) inhibit functioning of the CPT transporter (53, 54), while a large array of viruses also suppress MTP activity (55, 56). At the same time poxviruses, flaviviruses, and some other viruses enhance β-oxidation (29).

## IMPRINT OF CONVENTIONAL CULTURE MEDIUM ON CELL METABOLISM

The most widely used DMEM composition contains 25 mM glucose, whereas normal glucose levels in human blood are within a range of 4.91 ± 0.37 mM (57). Interestingly, the first formulation of DMEM had 5.5 mM glucose, which reflects the physiological level (20). Currently, this is referred to as low-glucose DMEM. The rationale for usage of such high concentration of glucose was that scientists work mainly with transformed or cancer cell lines often displaying a Warburg effect (58). The Warburg effect is frequently described as featuring increased glucose consumption and dependence on anaerobic glycolysis rather than oxidative phosphorylation in mitochondria. The latter is not correct, as many tumors do not exhibit decreased mitochondrial respiration. So, the Warburg effect is rather a disconnection of the glycolysis pathway from the TCA cycle (59), which feeds respiration complexes with succinate, NADH, and reduced flavin adenine dinucleotide (FADH_2_) (60). Notably, very recently it has been demonstrated that glycolysis and in particular the step of lactate production also promote the TCA cycle by providing oxidative equivalents via production of NAD^+^ (61).

Several years ago, K. Birsoy’s group reported that increased consumption of glucose by cancer cells leads not to an increase but to a decrease in its level in the tumor milieu (62). In their experimental work, they used an elegant approach of keeping the glucose level at 0.5 mM with constant replenishment of this nutrient via a Nutrostat apparatus. The decrease in glucose levels affected mitochondrial respiration and, therefore, allowed the researchers to register different sensitivities of Jurkat cells to biguanides, including the antidiabetes drug metformin.

Pyruvate is the end product of glycolysis as well as of several transamination reactions. It can be imported into mitochondria by the mitochondrial pyruvate transporter (mitochondrial pyruvate carrier [MPC]) and later be used for the synthesis of AcCoA. To ensure efficient cell growth (63) and virus replication (64), pyruvate is often added to culture medium at millimolar concentrations, since in contrast to glutamine, its use by the TCA cycle is not accompanied by production of toxic ammonia. As its levels in human plasma are 0.035 to 0.024 mM (57), adding millimolar concentrations is nonphysiological. It could be speculated that providing such extra high concentrations of pyruvate, the end product of glycolysis, may saturate the TCA cycle and mitochondrial respiration with substrates making glycolysis efficiency not very important for cells and thus not allowing researchers to explore the impact of viruses on glucose metabolism. Moreover, Vande Voorde et al. demonstrated that at millimolar levels pyruvate can stabilize hypoxia-inducible factor 1α (HIF1α), triggering a pseudohypoxic phenotype (24).

Respiratory activity of mitochondria depends not only on mitochondrial density, morphology, and concentrations of the substrates of the respiratory complexes but also on the assembly of respiratory complexes into “supercomplexes,” or respirasomes (65). It was clearly shown that association of complexes I, III, and IV leads to increased consumption of oxygen and ATP production. Supercomplex assembly is known to promote tumorigenesis by conferring metabolic adaptation of tumor cells (66). Suppressed glycolysis stimulates respiration by enhanced assembly of supercomplexes, as clearly demonstrated by Balsa et al. in cells cultivated in the presence of reduced levels of glycose or its substitute galactose (67). The dissected mechanism by which the cell facilitates formation of respirasomes involves activation of the ATF4 transcription factor that induces expression of the supercomplex assembly factor 1 (SCAF1/Cox7A2L) (67). Interestingly, this factor is activated in response to amino acid starvation via the Gcn2 protein kinase, specifically during exhaustion of asparagine (68). Indeed, Christofk’s group demonstrated that exogenous Asn not only suppresses ATF4 signaling but inhibits mitochondrial respiration to promote nucleotide biosynthesis (69). It is worth noting that depletion of Leu (70), Trp and Gln (71), and Met and Cys (72) may also activate ATF4 signaling.

Another metabolite that affects metabolism is arginine. In human plasma its level is estimated as 64 μM, while in DMEM, RPMI medium, and DMEM–F-12 its levels reach 398, 1,148, and 700 μM, respectively. Normally, arginine is converted within the urea cycle either into the nonproteinogenic amino acid ornithine via arginase or into citrulline, which is another metabolite of the urea cycle, by nitric oxide (NO) synthases (73). Using an isotope tracing approach, Tardito’s group observed that in triple-negative breast cancer (TNBC) cell lines arginine at an ultrahigh concentration is converted into argininosuccinate by argininosuccinate lyase (ASL), i.e., via the reverse reaction (24). This puts into question the relevance of production in classical media of such metabolites as NO and ornithine, the precursor for biogenic polyamines (74, 75).

Finally, the component in culture medium that has the most unpredictable impact on cellular processes is fetal bovine serum (FBS). As serum-free media have very limited usage in virology and in molecular biology, most researchers use FBS to provide cultures with essential growth factors. However, FBS also supplies low-molecular-weight nutrients at undefined concentrations. In response to that, some groups (for example, see reference 76) use not convenient but dialyzed FBS, devoid of polar metabolites, and HPLM is an example (23). In contrast, Plasmax developers used standard FBS but decreased its quantity from 10 to 2.5% without affecting cell viability and growing rates (24). Notably, 2.5% FBS is often used for cultivation of Vero cells in the context of propagation of a variety of viruses, including SARS-CoV-2 (77).

## DIFFERENCES IN CELL METABOLISM BETWEEN CELLS CULTIVATED IN CONVENTIONAL MEDIA AND CELLS CULTURED IN PLASMA-LIKE MEDIA

Substitution of Plasmax or HPLM for classical media leads to changes in several key metabolic processes due to the availability of nutrients at physiological levels and because a much wider range of metabolites is present in the latter medium (>70 organic components).

One of the major differences between cells cultured in classical media and those cultured in novel, physiological, media is the different input of central metabolic pathways in maintaining the TCA cycle and mitochondrial respiration. Our and other groups found that Plasmax medium ensures elevated respiratory activity in an array of cell lines used for both virus research (Vero E6, Huh7.5, A549, and HeLa) (78) and for studies mostly in connection with their tumor background (LNCaP for prostate carcinoma, MCF7 for breast carcinoma, SaOS2 for osteosarcoma, A375 for melanoma, and SW620 for metastatic colon carcinoma [79, 80]). The most pronounced difference was observed in respect to hypoxia. As oxygen availability is reduced in tissues compared to the standard laboratory settings (81), and mitochondria play significant roles in replication and pathogenesis of different viruses (82, 83), this finding can have a critical importance for the virology field. A recent report by Torres-Quesada et al. revealed that cancer cells in HPLM compared to those in standard media showed mild uncoupling of respiration and signs of increased mitochondrial density (79). At the same time, we must mention that in the initial paper by Cantor et al. an opposite effect, i.e., a decreased level of respiratory activity, was described for P12-Ichikawa and SUDHL4 cell lines (23).

Increased levels of respiration were not due to enhanced mitochondrial biogenesis, as revealed by assessing their total mass and maturation (77). However, cells cultivated in Plasmax medium displayed a higher ratio of mitochondria fused into vast networks. It is a well-established fact that increased fusion of this organelle correlates with higher respiratory activity (84). Fused mitochondria can also exhibit increased levels of supercomplexes, although the latter could result also from crista remodeling (85). But it is unlikely that the increased activity of these organelles in physiological media was due to assembly of such respirasomes: in our hands cells in Plasmax did not upregulate expression of the SCAF1/Cox7A2L factor that is considered a master regulator of supercomplex formation (77). However, to our knowledge no one has properly addressed this question by quantifying respirasomes on blue native gels.

Increased availability of the substrate for the TCA cycle and respiratory complexes could be another factor in higher oxygen consumption of cells in physiological media. Indeed, the Krebs cycle is fed not only by pyruvate produced by glycolysis but also by (i) its formation from lactate, (ii) AcCoA generated from the fatty acid oxidation pathway, and (iii) glutamine that is converted into α-ketoglutarate via glutamate and some other pathways. Although for lung adenocarcinoma A549 cells enhanced glycolysis was observed in Plasmax medium, the opposite was seen for HeLa, Vero E6, LNCaP, MCF7, and SaOS2 cell lines, while no differences between classical and physiological media were noted for Huh7.5 cells (77). Obviously, these data did not correlate with respiratory activity. Recent data also clearly demonstrated that under reduced oxygen levels glucose utilization is shifted from the TCA cycle toward production of lactate and toward the pentose phosphate pathway, at least in bone marrow stem cells (8). Therefore, it is not likely that increased respiration was due to enhanced production of the carbon donor substrates by glycolysis.

In contrast, physiological media can enhance mitochondrial respiration by providing substrates to the TCA cycle or by increasing input to the cycle of pathways other than glycolysis. As such, lactate, carnitine, and citric acid can be key players. It is widely known that blood plasma contains lactate at millimolar concentrations. Moreover, exercise, which enhanced glucose conversion to lactate with its efflux to the bloodstream, will additionally increase levels of the latter. The highest levels of lactate are found in the tumor milieu, where it can reach from 10 mM to 20 to 40 mM (86). Lactate has been considered an end product of glycolysis, whereas in recent years Rabinowitz’s group (87, 88) provided convincing data that it can be adsorbed by many tissues and be reconverted to pyruvate, thus feeding the TCA cycle. There are several reports of increased respiration of cells cultivated in classical media with additional lactate supplementation (89). Carnitine does not provide carbon moieties to the Krebs cycle. Instead, it is used for influx of fatty acid β-oxidation products into mitochondria via the carnitine palmitoyltransferase (CPT1) transporter. We showed that cells in Plasmax exhibit higher sensitivity to the CPT1 inhibitor etomoxir, suggesting that carnitine is indeed a key molecule in the regulation of cell metabolism in Plasmax (77). Finally, it is tempting to speculate that additional feeding of the TCA cycle is provided by citrate, as earlier this year Finley’s group (90) published an outstanding paper describing the noncanonical Krebs cycle that plays a role in defining cell pluripotency and (un)differentiation. Specifically, they showed that citrate can be exported from mitochondria to the cytoplasm and be converted into fumarate by the ATP citrate lyase (ACLY) and later to oxaloacetate (OAA) that is then channeled back to mitochondria. With these data one can assume that citrate, present in HPLM and Plasmax, can not only support fatty acid biosynthesis due to production of AcCoA by ACLY in the cytoplasm but also give rise to OAA in mitochondria. However, a report from Tardito’s group demonstrated that mesenchymal stem cells in Plasmax as well as in DMEM produce and secrete lactate (91). They also showed that a majority of citrate produced from AcCoA is exported to the cytoplasm, thus supporting evidence for a “Finley cycle.” However, one should note that such a phenotype could be a feature of stemness, and its occurrence in differentiated cells remains to be examined in future.

Another difference in cell metabolism between cells cultivated in conventional media and those cultivated in physiological media is an inhibition of UMP synthase (UMPS), an enzyme catalyzing biosynthesis of UMP which also serves as a precursor for CMP (23). As a result, cells maintained in HPLM exhibit accumulation of early metabolites of a *de novo* pyrimidine nucleotide biosynthesis pathway: carbamoyl aspartate, dihydroorotate, orotate, and orotidine. This effect is solely dependent on the presence of uric acid in the medium that acts as a UMPS inhibitor. This effect has an important therapeutic implication: cells in classical and physiological media exhibit different sensitivities to the antitumor drug 5-fluorouracil. Since inhibitors of nucleotide biosynthesis are known to display antiviral activity, as shown for example for the enzyme CAD (92), as CAD is a trifunctional enzyme, and this abbreviation stands for carbamoyl-phosphate synthetase 2, aspartate transcarbamylase, and dihydroorotase or dihydroorotate dehydrogenase (93) of pyrimidine biosynthesis or IMP dehydrogenase (IMPDH) of GTP synthesis (94), their efficacy should be reevaluated in physiological media.

Finally, physiological media were shown to affect biosynthesis of amino acids and channeling of glucose metabolites from glycolysis into the pentose phosphate pathway for production of nucleotides. Using isotope flux analysis, Cantor et al. showed that Plasmax stimulated biosynthesis of asparagine and valine but suppressed production of alanine (23). Partially contrary results were published by Taurino et al., who showed increased production and secretion of Ala, Asn, Pro, and uridine in DMEM compared to those in Plasmax, which was in line with their presence in the physiological medium (88). HPLM also decreased the ratio of ^13^C-labeled fructose-6-phosphate (which proceeds to distal stages of glycolysis) and glucose-1-phosphate (the first metabolite of the PPP) from the labeled glucose (23, 88). This either can indicate suppressed glycolysis or suggests enhanced nucleotide biosynthesis. Interestingly, as control of glucose-6-phosphate flux into either glycolysis or the PPP is maintained by hexokinase 1 and its intracellular localization (cytoplasm or on the outer membrane of mitochondria) (95), it is really tempting to speculate that physiological media can target this enzyme which is critical for at least HIV-1 replication in macrophages (96).

## IMPACT OF MEDIUM ON CELL REDOX STATUS

Redox status of a host cell depends on rates of production of reactive oxygen species (ROS) and cellular antioxidant capacity. ROS scavenging is mainly achieved by two families of highly active peroxidases, peroxiredoxins and glutathione peroxidases, as they have high affinity to H_2_O_2_ and organic peroxides (97, 98). Neutralization of ROS leads to oxidation of these enzymes with subsequent recycling by thioredoxin or glutaredoxin systems. Importantly, both thioredoxin and glutaredoxin reductases, the end-stage recycling enzymes, utilize NADPH as a reducing equivalent (99). In their turn, oxidized NADP^+^ molecules are reduced via the pentose phosphate pathway that gives rise to nucleotides, the building blocks for nucleic acid synthesis, with glucose-6-phosphate dehydrogenase (G6PD) being the major NADPH-generating enzyme (100, 101). Alternatively, NADP^+^ could be recycled via folate metabolism, isocitrate dehydrogenases 1 and 2 (IDH1 and IDH2), or malic enzyme of anapleurotic pathways of central carbon metabolism, nicotinamide nucleotide transhydrogenase (NNT), and several other enzymes (102). IDH2 and G6PD are the major NADPH-producing enzymes in mitochondria and the cytoplasm, respectively. So, changes in nutrient supplies resulting from a shift from classical to plasma-like medium can affect the NADPH pool and, as a result, the functioning and capacity of ROS-scavenging systems.

Redox status also depends on the NAD^+^/NADH ratio, which strongly depends on concentrations of pyruvate and lactate, as conversion of pyruvate to lactate by lactate dehydrogenases is accompanied by a stochiometric reduction of NAD^+^. So, addition of pyruvate to classical media shifts redox status to the reduced, whereas the presence of lactate at millimolar concentration in Plasmax or HPLM changes the NAD^+^/NADH ratio toward the oxidized forms. NAD^+^ itself also regulates redox states of the cells by modulation of sirtuins 2, 3, and 5 (Sirt2, Sirt3, and Sirt5, respectively) (103–105) that act as sensors of the NAD^+^/NADH ratio (106). Sirt3 and Sirt5 activate IDH2, which is the major NADPH-producing enzyme in mitochondria, by its deacetylation, which further increases the intracellular activity of this enzyme (107, 108). Sirt2 and Sirt5 activate G6PD via deacylation (108, 109). Indeed, cells cultivated in HPLM demonstrated altered oxidized glutathione (GSSG)/glutathione (GSH) and NAD^+^/NADH ratios, although the precise effect was cell line dependent (23). Moreover, a recent study by Wang et al. demonstrated that an enhanced glycolysis and pentose phosphate pathway led to increased production of NADH that fed mitochondrial respiratory activity (61). Therefore, it is logical to assume that this would lead to enhanced production of superoxide anions due to electron leakage from the electron transfer chain.

Mitochondria are indeed one of the major sources of ROS in a cell. As discussed in the previous section, Plasmax does not change the mass of mitochondria but enhances their fusion into network structures and enhances their metabolic activity. Therefore, it is not surprising that cells in Plasmax demonstrate increased rates of ROS production, as revealed by redox-sensitive dyes 2′,7′-dichlorodihydrofluoresceine diacetate (DCFH2DA) and superoxide-anion-specific dihydroethidium (DHE) or its mitochondrially targeted MitoSOX analogue (77). Also, Plasmax ensured more pronounced ROS production in various cell lines infected with respiratory viruses (influenza virus and SARS-CoV-2) or hepatitis C viruses, indicating that physiological media are suitable for redox biology studies (77). It is tempting to speculate that lower levels of ROS production in response to viral infections in cells cultivated in classical media could be due to some antioxidant effect of the latter that can inhibit or even prevent virus-induced oxidative stress. Indeed, Long and Halliwell described ROS scavenging by pyruvate that is often added to culture medium (110). Though in our group pyruvate-free classical media are used, the antioxidant effect could be mediated by other components such as FBS, for instance (111).

One of the key components of Plasmax medium that affects cellular antioxidant defense capacity is selenite. It is required for expression of functional selenoproteins, and glutathione peroxidases in particular. It has been clearly demonstrated by Tardito’s group that the presence of selenite protects cells against ferroptotic cell death, occurring at extremely low cell density (24). Ferroptosis occurs upon downregulation or inhibition of the lipid peroxide scavenger glutathione peroxidase 4 (GPx4) or blockage of cysteine import proteins leading to exhaustion of glutathione pools and enhanced lipid peroxidation (112). Indeed, addition of selenite to standard culture medium leads to markedly increased GPx4 expression (24). So, the cells in Plasmax on one hand and in classical media or HPLM on the other demonstrate different susceptibilities to lipid peroxidation (24) and presumably to inducers of ferroptosis and viruses that modulate the expression of GPx4 and/or cysteine metabolism.

Nutrient levels significantly modulate expression of several ROS-producing enzymes such as NADPH-oxidases (NOX). NOX1 to -5 and their analogues DUOX1 and -2 are membrane multisubunit proteins that generate superoxide anions of hydrogen peroxide during oxidation of NADPH (113). Among them, NOX1 and NOX4 are the isoforms expressed in mammalian cells in a variety of organs including colon and kidney (114, 115). Interestingly, increased levels of glucose in culture medium significantly upregulate expression of both enzymes, leading to enhanced ROS production (116). Additionally, elevated levels of NADPH, i.e., the substrate of NOX/DUOX family members, should additionally stimulate generation of reactive oxygen species. As several groups, including ours, showed that some viruses, including hepatitis C virus (HCV) (78, 117, 118), promote ROS production in infected cells by upregulation of Nox1 and Nox4, it could be important to reevaluate these data in the setting of physiological media that should not themselves affect their expression.

## PLASMA-LIKE MEDIA FOR THE STUDY OF VIRUSES

So far there is only a single report, published by our group, that describes the use of plasma-resembling Plasmax medium for cultivation of viruses (77). We focused mainly on hepatitis C virus (HCV), influenza A virus (IAV), and SARS-CoV-2 replicating in human hepatoma Huh7.5 cells, lung adenocarcinoma A549 cells, and Vero E6 cells, respectively. In all cases the cells cultivated in Plasmax supported replication of these viruses, albeit at lower levels and with slower kinetics than those of cultures in standard media. For HCV, using the subgenomic replicon model, we showed that the decreased levels of replication were due to suppressed central stages of the virus life cycle (i.e., replication/translation). Similar experiments were also carried out with enteroviruses including coxsackie B3 and B5 viruses, poliovirus, Newcastle disease virus (NDV), and vesicular stomatitis virus (VSV) in Vero E6 and HeLa cells: decreased replication rates were observed only for NDV and poliovirus type 3, whereas for other viruses no impact of the medium was registered. So, the changes in replication efficiency in response to plasma-like media could be pathogen specific and probably related to replication kinetics.

One of the possible mechanisms by which Plasmax may decrease rates of replication of RNA viruses could be enhanced mitochondrial respiration and the linked TCA cycle. Recently, Lee et al. (119) reported that activation of the TCA cycle by addition of pyruvate or fatty acids as carbon sources of nicotinamide riboside as a source of oxidative equivalents via NAD^+^ restrained replication of murine hepatitis virus, a surrogate model of coronaviruses. At the same time, we should admit that other, yet-unidentified, mechanisms could also underlie the reduced replication capacity of the viruses in plasma-like media.

## CAVEATS IN USAGE OF PLASMA-LIKE MEDIA

Although plasma-resembling media are more physiological than the classical media, their usage can lead to several possible artifacts that should be avoided. First, substitution of Plasmax or HPLM for standard medium does not immediately normalize biological processes in cells. Kinetic analysis revealed that cell morphology starts to change after medium substitution only after 3 days, as exemplified in the case of hepatoma Huh7.5 and carcinoma HeLa cell lines (77). Moreover, analysis of HCV replication kinetics in cells harboring a subgenomic replicon revealed that it takes up to 6 days for the virus RNA level to reach a plateau after a gradual decrease that started at day 2 to 3. So, adaptation of cells to physiological media during a week is required, while a shorter incubation may not be sufficient to induce the desired changes.

Second, prolonged cultivation of cells in Plasmax and presumably in HPLM may enhance *de novo* serine biosynthesis by upregulation of Phosphoglycerate dehydrogenase (PHGDH) and Phosphoserine aminotransferase (PSAT), and phosphoserine phosphatase (PSPH) expression, as well as of asparagine synthetase. Indeed, this has been recently described by Vousden’s group and ours (77, 120). As expression of these genes is controlled by the ATF4 factor (69, 121), the master regulator of the integrated stress response, such change could be due to exhaustion of some amino acids. Gardner et al. also revealed that amino acid and glucose exhaustion in Plasmax is associated with the unfolded protein response (122). Regular medium replacement to prevent nutrient starvation completely prevented upregulation of expression of all the above-mentioned genes (77, 122).

Finally, since Plasmax contains selenite in its composition, induction of ferroptosis may be more difficult to study. Notably, for investigation of this type of cell death HPLM could be more appropriate, as it lacks selenite.

## LIMITATIONS OF THE USE OF PLASMA-LIKE MEDIA

The rationale for the development of plasma-like media is that cells should be cultivated in the presence of a wide spectrum of nutrients at physiological concentrations. As such, the composition of blood plasma was used as a standard. However, several key parameters will need to be further refined in the future. First, different metabolomic techniques such as liquid chromatography-mass spectrometry (LC-MS), gas chromatography-mass spectrometry (GC-MS), and nuclear magnetic resonance (NMR) produce heterogeneous results regarding quantification of some nutrients (57), leading to discrepancies in what levels should be regarded as physiological. A second caveat is that metabolite compositions of blood serum and plasma are also quite different (123). Third, in most cases concentrations of nutrients correspond to serum of the blood taken from peripheral veins, while in arteries blood composition is known to be different (124, 125). Fourth, *in vivo* many cell types are not exposed to serum directly but are separated from it by endothelia and other cells that also utilize nutrients and secrete additional metabolites while trafficking nutrients from the bloodstream to the surrounding tissues (126). Moreover, oxygen levels also dramatically differ in various cells of the same organ (127, 128). As a result, a metabolic zonation exists within a tissue that represents a gradient of nutrients depending on distance from blood vessels and its type (vein or artery) (128, 129). So, cells of the same type in different parts of the tissue may have different metabolic traits. An example is a publication from Hibner’s group, who described zonation in the liver in the context of HCV infection (130). In the perivenous zone uninfected hepatocytes exhibit a glycolytic phenotype, actively synthesize triglycerides and lipids, and utilize ammonia to produce glutamine (127, 128). In contrast, within the periportal zone, hepatocytes exhibit oxidative phosphorylation, lactate and ammonia are used by gluconeogenesis and the urea cycle, respectively, and fatty acid β-oxidation is active. This could be further expanded by speculating about possible differences between nutrient and oxygen supplies of the central nervous system and lungs and those of other tissues of the organism, keeping in mind the existence of systemic, pulmonary, and coronary circuits of blood circulation. So, different organs may be exposed to different nutrient compositions and concentrations. And the metabolic phenotype of cells that are distant from the bloodstream drastically differs from that of proximal cells.

Mucosal epithelial cells represent one of the most interesting types of cells for virologists, keeping in mind that they are often infected by a variety of pathogens such as herpes simplex viruses I and II (131, 132), respiratory viruses (133, 134), viruses that enter mucosal cells in the gastrointestinal tract, including rotavirus and SARS-CoV-2 (133, 135) and many others, and viruses that enter in conjunctiva, etc. (136). These cells are directly exposed to oxygen (in the case of the respiratory tract) or nutrients (in the case of the gut), which should definitely have a profound imprint on their metabolic landscape. Moreover, within the gastrointestinal tract there is a gradient of nutrients, implying that cells of different parts of the colon may be exposed to different levels of nutrients (137). Currently, different vendors supply specialized media for a particular type of cells such as BrainPhys or NeuroBasal for neural cells, alveolar epithelial cell medium, endothelial cell growth medium, and some others. Most of them have optimized concentrations of inorganic ions, a reduced FBS concentration, or a chemically defined composition to avoid usage of animal serum. But it is obvious that while these media support cell growth, their composition is still far from physiological. All these parameters underline the necessity to develop modifications of physiological media for cultivation of specific types of cells.

## FUTURE DIRECTIONS

Until now, there has been no systematic analysis of metabolic changes occurring during viral infection in cells maintained in plasma-resembling media. Based on our preliminary data, we can propose that classical media that contain several components in artificially high concentrations may saturate nutrient import, thus blunting any effects of viruses. If so, conducting research in plasma-like media can allow detection of changes in catabolic and/or anabolic pathways of a host cell.

One of the most notable changes in mammalian cells occurring in Plasmax medium is a dramatic decrease in total lysosomal mass (77). Reasons for this decrease remain unknown. As viruses heavily interfere with functions of lysosomes and the processes they are involved in (autophagy, mitophagy, etc.), one cannot exclude that the real effect of viruses on lysosomes may differ from that observed in cell lines maintained in classical media.

Another direction that merits further studies is the reevaluation of an imprint of viruses on host cell metabolism. As an example, HCV, herpesvirus, and other viruses were previously reported to modulate the glutaminolysis pathway and rely on these changes (34, 36, 138, 139). However, the majority of cancer cell lines demonstrate upregulated glutaminolysis and dependence of cell growth on expression/activity of glutaminase (140). However, such dependence of cells on glutamine for many cancer cell lines was shown to be an artifact of *in vitro* 2D cell lines maintained in nonphysiological media (141), whereas in plasma-like medium, in three-dimensional (3D) cultures, or in *in vivo* systems this dependence may be absent.

The third direction for the use of plasma-like media in virus research is to investigate pathogen-induced oxidative stress. As cell metabolism is tightly linked with redox pathways, viruses may affect different ROS-producing enzymes and redox-sensitive transcription factors in different media.
